# Analysis of Differential Gene Expression and Novel Transcript Units of Ovine Muscle Transcriptomes

**DOI:** 10.1371/journal.pone.0089817

**Published:** 2014-02-26

**Authors:** Chunlan Zhang, Guizhi Wang, Jianmin Wang, Zhibin Ji, Fei Dong, Tianle Chao

**Affiliations:** 1 Shandong Provincial Key Laboratory of Animal Biotechnology and Disease Control and Prevention, College of Animal Science and Veterinary Medicine, Shandong Agricultural University, Taian, China; 2 College of Biological and Agricultural Engineering, Weifang University, Key Laboratory of Biochemistry and Molecular Biology in Universities of Shandong, Weifang, China; Wageningen UR Livestock Research, Netherlands

## Abstract

In this study, we characterized differentially expressed genes (DEGs) between the muscle transcriptomes of Small-tailed Han sheep and Dorper sheep and predicted novel transcript units using high-throughput RNA sequencing technology. Gene Ontology (GO) and Kyoto Encyclopedia of Genes and Genomes (KEGG) analyses showed that 1,300 DEGs were involved in cellular processes, metabolic pathways, and the actin cytoskeleton pathway. Importantly, we identified 34 DEGs related to muscle cell development and differentiation. Additionally, we were able to optimize the gene structure and predict the untranslated regions (UTRs) for some of the DEGs. Among the 123,678 novel predicted transcript units (TUs), 15,015 units were predicted protein sequences. The reliability of the sequencing data was verified through qRT-PCR analysis of 12 genes. These results will provide useful information for functional genetic research in the future.

## Introduction

Sheep are important domestic animals in China, and mutton accounts for a large percentage of the meat consumed. A growing market demand for mutton has promoted sheep breeding programs. Therefore, the identification and characterization of the genes that regulate muscle growth in sheep is a pressing problem. In recent years, studies of gene expression in sheep mainly used the human GeneChip [1], a cattle cDNA microarray [2]–[5], and the first sheep oligo DNA microarray [6]. Microarrays can be used to effectively examine the differences in known expressed genes, but they have some limitations; particularly, microarrays fail to recognize the lowly expressed genes, sequence variations, and novel transcript units.

Next-generation sequencing provides a platform for measuring gene expression in a manner that is more sensitive than traditional microarray hybridization experiments [7]. It has become widely used in recent years to investigate the DEGs and novel transcript units in all types of organisms, including rice [8], pigs [9], cows [10], and others. Currently, there are several studies that describe the transcriptomes of bone [11], skin [12], and ovary [13] in sheep using high-throughput sequencing. However, to our knowledge, there is very little transcritptome information related to muscle in sheep. To completely understand the complexity of gene expression changes between sheep will help to investigate the molecular genetic mechanism of muscle growth. In our previous study, we reported discovered DEGs and novel TUs in the muscle transcriptome of Dorper (DP) and Small-tailed Han sheep (SH), but not an in-depth analysis on them [14]. In order to search genes closely associated with muscle growth, here, we present a further analysis of these DEGs and novel TUs. This information will provide the basis for our future study of genes.

## Results

### Differentially Expressed Genes (DEGs) Analysis

Construction of the muscle transcriptome libraries, sequencing, and discovering of DEGs were summarized previously [14]. Of the 1,300 DEGs, 24 and 17 highly expressed genes (RPKM ≥500) were identified in the DP and SH sheep, respectively. Among these genes, 11 were identical between the two samples (Table S1 in File S1). Subsequently, the UniProt protein names of these 11 highly expressed genes were identified by querying the bioDBnet (http://biodbnet.abcc.ncifcrf.gov/db/db2db.php#biod) database. We found that these genes were mainly related to muscle myofibers (myosin, troponin, myoglobin, and tropomyosin); some enzymes (NAD^+^, PDK4, and protein phosphatase); and a variety of other proteins, such as heat shock 70 kDa protein, ankyrin repeat proteins, and SOCS box protein 5. Six genes related to muscle myofibers were down-regulated in the SH library.

### Gene Ontology (GO) Functional Enrichment Analysis for the DEGs

**Figure 1:**
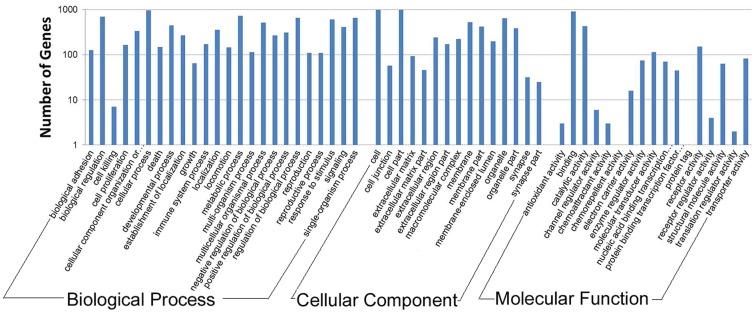
Gene ontology classification for the DEGs. The figure shows the GO enrichment of the differentially expressed genes in terms of molecular function, cellular components, and biological processes.

To further investigate the biological importance of the differentially expressed genes, we determined the functional categories of each gene by querying the GO database (http://www.geneontology.org/GO.database.shtml). The 1,300 DEGs were categorized into 56 functional groups based on sequence homology, which included 24 biological process, 15 cellular component, and 17 molecular function annotation. Among these functional groups, there was annotation of 82.00% (1,066/1,300), 87.46% (1,137/1,300), and 82.08% (1,067/1,300) of the DEGs, respectively (Figure 1).

Cellular processes (GO: 0009987), with 963 genes, was the dominant second category of the biological process group. Cell (GO: 0005623) and cell parts (GO: 0044464) occupied the main portion of the cellular component category, with 979 and 984 genes, respectively. However, in the molecular function category, the dominant category was binding (GO: 0005488), which consisted of 903 genes. We also observed a high percentage of genes in the biological process group that were involved in metabolic processes (GO: 0008152; 728 genes) and biological regulation (GO: 0065007; 698 genes).According to the results of the GO analysis, there were approximately 10 processes, which included 34 genes, related to muscle cell development and differentiation (Table S2 in File S1). In the SH library, genes encoding myofiber proteins (myosin regulatory light chain 2, myosin heavy polypeptide 6, and myocardin) and a related kinase (myosin light chain kinase 3) were down-regulated; however, genes encoding an integrin signaling molecule (integrin alpha 8), collagen alpha 1 chain, and tenascin C were up-regulated. Additionally, the regulating factors IGFBP5, VEGFA, and MEF2A were down-regulated, whereas TGFBR3 was up-regulated in the SH sample.

### KEGG Pathway Analysis

To further understand the function of these DEGs, we mapped them to terms in the KEGG database (http://www.genome.ad.jp/kegg/). There were 1,152 DEGs identified in 237 pathways with a KEGG pathway annotation (Table S3 in File S1). Metabolic pathways (114 genes), ECM-receptor interaction (66 genes), and the regulation of the actin cytoskeleton (53 genes) were the pathways with the most genes. We believed that these pathways were significant during the growth of sheep, in particular metabolic pathways. The second largest category was ECM-receptor interaction (ko04512); 51 genes involved in this pathway were up-regulated, and 28 genes were down-regulated in the SH library (Figure S1 in File S1). A large number of genes were involved in the regulation of the actin cytoskeleton pathway (ko04810); 21 genes were up-regulated, and 18 genes were down-regulated in the SH library (Figure S2 in File S1). Regret, it has no graph of metabolic pathway in the KEGG. Some vital signaling pathways, importantly, including PPAR, MARP, TGF-β, VEGF, and Jak-STAT, were represented.

### Optimization and Extension of the DEGs

To define the 5′ and 3′ gene boundaries more precisely, we investigated the upstream and downstream regions of the 1,300 DEG transcripts. In the SH and DP libraries, 644 and 569 DEGs, respectively, were extended (Table S4 in File S1). Among these DEGs, 808 genes were extended at the 3′ end, 261 genes were extended at the 5′ end, and 144 genes were extended at both ends. Between the two samples, we identified 183 genes that were extended at both ends.

### UTR Prediction of the DEGs

In this study, we investigated the possible UTRs of the DEGs using the results of our extension analysis (Table S5 in File S1). 310 genes with complete ORFs were predicted UTRs. Among these genes, 58 (18.71%) and 155 (50.00%) had only the 5′ UTR or the 3′ UTR, respectively. A total of 97 (31.29%) genes possessed both the 5′ and 3′ UTRs. We were able to predict UTRs of 37 DEGs with incomplete ORFs. Among these, 2 (5.41%), 15 (40.54%), and 20 (54.05%) genes were predicted to have the 5′ UTR, the 3′ UTR, or both ends, respectively.

### Protein-protein Interacting Network of the DEGs

**Figure 2:**
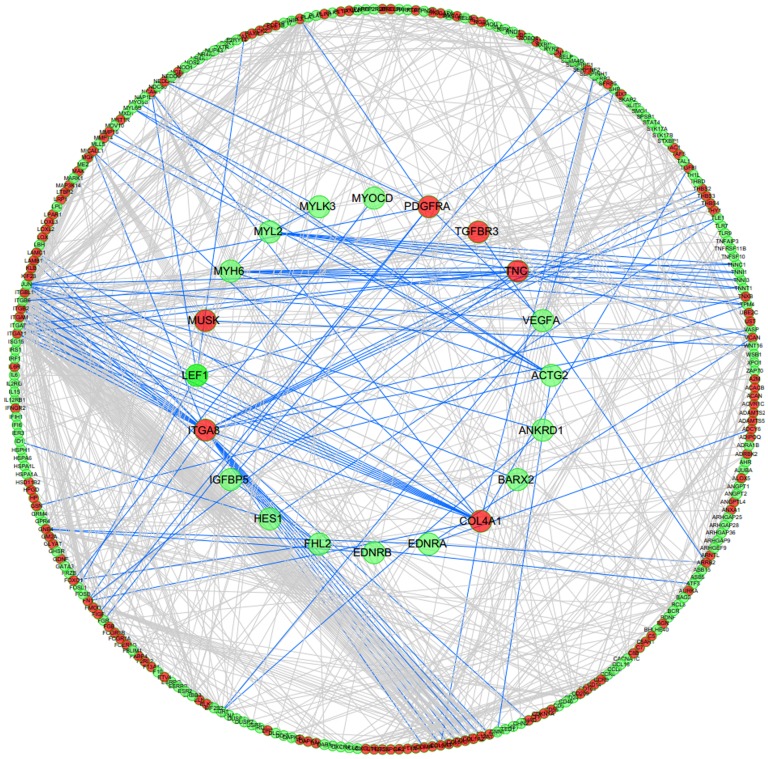
Protein-protein interaction network of the significantly differentially expressed genes. The sketch represents the interaction network constructed for 1,009 protein-coding DEGs. The red and green dots represent the up-regulated and down-regulated genes, respectively. The 20 proteins related to muscle cell development are underlined in the center of the circle. The lines represent the interactions between the gene products.

We also examined potential protein-protein interactions of the 1,300 DEGs. Because there is no functional interaction network for sheep, we mapped these genes to the human Reactome data set to identify functional interaction (FI) networks, according to the method of Jinsil Kim [15]. Before mapping, the IDs of the 1,300 DEGs were converted into gene symbols for the encoded proteins using bioDBnet software; however, only 1,009 genes were given symbols (Table S6 in File S1). We used the edge betweenness algorithm [16] to identify the functional modules in the network. Of the 1,009 total DEGs and 34 DEGs that were related to muscle cell development and differentiation, only 299 and 20 proteins were included in the human Reactome database, respectively (Table S2 in File S1). We then studied the interaction of the 20 proteins related to muscle cell development and differentiation with other proteins. As shown in Figure 2, integrin (ITGA8) and collagen (COL4A1) had numerous interactions. However, myosin, troponin, and other regulating factors (IGFBP5, TGFBR3, and VEGF) that are closely related to growth and development had few interactions with other proteins.

### Characteristics of Novel Transcript Units

Based on the transcriptome data aligned with the sheep reference genome, 66,668 and 57,010 novel transcript units (TUs) were identified in the DP and SH libraries, respectively [14]. After removing the novel TUs without complete ORFs, we predicted 15,546 and 13,369 UTR sequences for the 7,462 and 6,936 novel TUs in the DP and SH libraries, respectively (Table S7 in File S1). In the DP sample, 7,416 novel TUs had both 5′ and 3′ UTRs, 69 TUs had only a 5′ UTR, and 645 had only a 3′ UTR. In the SH library, 9 novel TUs had 5′ UTRs, 31 had 3′ UTRs, and 6,896 had both 5′ and 3′ UTRs (Table S8 in File S1). Among these predicted novel transcripts, some encoded proteins, whereas others did not. For TUs with complete ORFs, we predicted 8,077 and 6,938 protein sequences, which accounted for 12.12% and 12.17% of the total novel TUs in the DP and SH libraries, respectively (Table S9 in File S1). The longest protein sequence was 3,061 amino acids in length, and 1,420 TUs had 100% similarity between the two libraries.

### Quantitative RT-PCR Validation of the Sequencing Data

**Figure 3:**
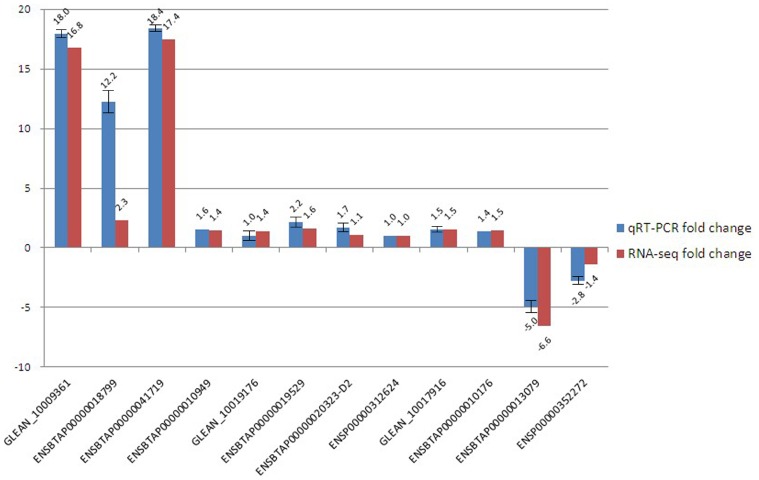
qRT-PCR validation of the expressed genes using the Illumina sequencing technology. For the 12 randomly selected differentially expressed genes, fold changes of DP/SH determined from the relative Ct values of using the 2^−△△CT^ method in qRT-PCR were compared to those detected by RPKM of DP/SH in RNA-seq. All Ct values were normalized to GAPDH and replicates (n = 3) of each sample were run.

To confirm the reliability of the high-throughput sequencing data, we used qRT-PCR to compare the expression levels. Among 18 genes randomly selected from 1,300 DEGs, 12 genes were validated, and 6 were not detected. Of the 12 detected genes, 4 were significantly differentially expressed, whereas 8 were not in RNA-seq. The primers used for each gene and GAPDH were summarized in Table 1. Fold change of DP/SH in qRT-PCR analysis and RNA-seq were summarized in Figure 3. Of the 4 significantly differentially expressed genes, GLEAN_10009361, ENSBTAP00000018799, ENSBTAP00000041719 and ENSBTAP00000013079, fold was correspondingly big (18.0, 12.2, 18.4 and −5.0 fold, especially) in qRT-PCR. But other genes showed corresponding lower expressing fold. In addition to ENSBTAP00000018799 had a bigger difference of fold change, the results of other 11 genes were in good agreement between RNA-seq and qRT-PCR.

**Table 1:** Primer sequences for qRT-PCR amplification of specific genes.

Gene ID	Forward primer (5′→3′)	Reverse primer (5′→3′)	Product size
GLEAN_10009361	gccaatctatttatccact	ctttagcacctgtctcatc	147 bp
ENSBTAP00000018799	agtcttgccttccttccc	agcagcagcgtgtaatgg	230 bp
ENSBTAP00000013079	gcttgccaatctatttatc	ctttagcacctgtctcatc	151 bp
ENSBTAP00000041719	ccaccacctgtccctcta	gcctccttgaactcctcc	116 bp
ENSBTAP00000010949	catcaacggaggcaacac	tgctcaaatcgggaacaa	122 bp
GLEAN_10019176	cccgagtgtatggagtgg	agcaaccgaggagtgaat	137 bp
ENSBTAP00000019529	gtgagggttggcacatta	cgagcagtaggtggtttt	221 bp
ENSBTAP00000020323-D2	cattgacaacagggtgcg	cttgcggttcttggagga	208 bp
ENSP00000312624	tgtcggaggagaactgtgagc	tggcgggagtgaagatgg	264 bp
GLEAN_10017916	gttcggttctgaccttga	ggtttgatgttggctgata	220 bp
ENSP00000352272	ttcgctgctcactaaccg	gcctccgacactgctctt	196 bp
ENSBTAP00000010176	aaatccaagacaaggagggc	tcgcagacgaagcaccag	137 bp
GAPDH	caccctcaagattgtcagc	cagtggtcataagtccctcc	117 bp

## Discussion

### Selection of Animals and the Reliability of the Sequencing Data

An analysis of the DEGs between sheep with different growth rates contributes to the identification of genes related to muscle growth. Here, we selected DP and SH sheep with same age and different growth rate as our study animals, whose apparence and characteristic were described as previous reports [14]. The different growth rates of muscle may be associated with differences in the regulation of gene expression between the two breeds.

Currently, high-throughput sequencing is widely used in many species. In our study, we randomly selected several differentially expressed genes using qRT-PCR to verify the accuracy and repeatability of the sequencing data. Of the 18 DEGs that were chosen, 12 (67%) were validated. Our inability to detect 6 DEGs may have been due to inappropriate primer design. In the 12 genes that were detected, the fold change of ENSBTAP00000018799 was in a larger difference between qRT-PCR and RNA-seq (12.2 and 2.3, especially), which might be caused by experimental error in our qRT-PCR (the difference was significantly among repetition). But the expression pattern of other 11 genes observed using qRT-PCR was consistent with that observed by RNA-seq. These results indicate that Illumina sequencing was reliable in our study. In addition, the lowly expressed genes, including GLEAN_10009361 (RPKM = 35.24) and ENSBTAP00000041719 (RPKM = 35.11), were verified using qRT-PCR. This result indicated that the Illumina sequencing was more sensitive than microarrays for estimating gene expression, especially for low-abundance transcripts [17].

### GO Function and KEGG Pathway Analysis of the DEGs

Of the 1,300 DEGs, 6 commonly expressed genes with high RPKM values that were related to myosin, troponin, myoglobin, and tropomyosin were down-regulated in the SH library. Due to the different growth rates of the Small-tailed Han sheep and the Dorper sheep (the growth of the former is slower than that of the latter), the 6 genes might be conserved and could be directly related to sheep muscle development.

As structural components of the extracellular matrix (ECM), collagens (COL4A1), integrin signaling proteins, and tenascin C exhibited the highest enrichment and up-regulation in the SH sheep in this study. These genes may be related to meat quality [18]. Genes that are related closely to growth and development, such as IGFBP5 and TGFBR3, were found to be down-regulated and up-regulated, respectively. This pattern is similar to the expression patterns observed in pig [19] and mouse [20]. TGFBR3 as a member of the TGF-β signaling pathway and can suppress cell proliferation and differentiation [21], [22]. In this study, TGFBR3 was up-regulated in the slower growing sheep, implying that TGFBR3 may negatively regulate muscle growth. On the other hand, IGFBP5 is a member of IGFBP family that specifically binds to IGFs and regulates many biochemical processes in the organism [21]. IGFBP5 has also been shown to regulate muscle cell proliferation [23] and myoblast differentiation [24]. The differences in the expression of IGFBP5 may be important due to the differences in muscle growth. All the genes mentioned here should be studied in detail in the future.

GO annotation and KEGG pathway analyses were used to obtain a better understanding of the DEGs. The GO enrichment analysis showed that a large number of the genes were involved in a wide variety of biological processes, such as cellular processes, binding, and metabolic processes. These results agree with previous gene expression studies conducted in pig [25] and suggest that different genes usually cooperate with each other to exercise their biological function. KEGG analysis showed that approximately 9.9% of the genes were involved in a metabolic pathway, and 5.7% were involved in ECM-receptor interaction. Additionally, 5 important signaling pathways (MAPK, TGF-β, Wnt, VEGF, and GnRH) were identified. These results indicate that a complicated gene network might be involved in muscle cell and tissue development or proliferation. The function of these transcripts should be further validated by additional biological analysis and experimental evidence.

### UTR Analysis of the DEGs

The identification and refinement of a large number of UTR boundaries would be useful for interpreting the regulatory factors that mediate gene expression and to improve existing gene annotation. To our knowledge, no transcriptome-wide UTR predictions have been previously performed in sheep. We were able to predict the UTRs of 310 reference DEGs according to their original sequence, and the UTRs of 37 DEGs could be predicted after extension, according our RNA-seq results.

### Protein-protein Interaction Analysis

One of the most intriguing findings came from our examination of potential interactions among the 1,300 DEGs genes; in these experiments, we compared the DEGs to human tissues using FI networks composed of sub-network modules enriched for specific gene categories and functional pathways. Proteins enriched in the extracellular matrix (ECM), such as integrin and collagen, had multiple interactions with other proteins. This result suggests a critical role of the ECM in processes involved in normal muscle biology, as well as other tissue biology. IGFBP5 is a member of the IGF signaling pathway, and VEGFA and TGFBR3 are members of the VEGF and TGF-β signaling pathways, respectively. These three pathways are involved in a wide range of cellular processes [26]–[28], which suggests that the diverse functions of the muscle can be regulated by several signaling pathways. The VEGF signaling pathway regulates vascular development in the embryo and new blood vessel formation (angiogenesis) [29]. The VEGF receptor, VEGFR, can also induce several cellular processes that are common to many growth factor receptors, including cell migration, proliferation, and survival [30]. Interestingly, these three regulatory factors had few interactions with other proteins; however, these results could be due to the selection of an inappropriate protein database.

## Conclusions

In this study, we examined the DEGs and novel transcripts of the muscle transcriptome in the Dorper and Small-tailed Han sheep using Illumina high-throughput sequencing platform. 1,300 DEGs between the two libraries were active in cellular processes, metabolic pathways, and the actin cytoskeleton pathway by GO analysis. A total of 34 DEGs were found to be related to muscle cell development and differentiation, and 20 proteins had obvious interactions with other proteins based on the protein-protein interaction network analysis. There were 1,152 DEGs identified in 240 KEGG-annotated pathways. Among the 123,678 predicted novel transcript units, 15,015 units were predicted to be protein-coding sequences. The expression patterns of 12 genes were confirmed by quantitative RT-PCR. The findings of this work may be a valuable resource for future in-depth studies related to muscle growth of sheep.

## Materials and Methods

### Ethics Statement

All animal experiments were approved by the Institutional Animal Care and Use Ethics Committee of Shandong Agricultural University (Permit Number: 2004006) and performed in accordance with the “Guidelines for Experimental Animals” of the Ministry of Science and Technology (Beijing, China). All surgery was performed according to recommendations proposed by the European Commission (1997), and all efforts were made to minimize suffering.

### Animals, Muscle, Analysis of DEGs and Novel TUs

Animals and biceps brachii collection, contruction of cDNA libraries, sequencing, analysis of DEGs and novel TUs were described as previous reports [14].

### Gene Ontology Functional Enrichment and KEGG Pathway Analysis for DEGs

All differentially expressed genes were classified into the categories of molecular function, cellular component, and biological process in the Gene Ontology (GO) database (http://www.geneontology.org/) using the Blast2GO software (version 2.3.5). An ultra-geometric test was applied, and significantly enriched GO terms in the DEGs were searched and compared to the genome background. The following calculation was used:
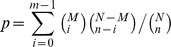



Where N is the number of genes annotated with GO terms, n is the number of DEGs in N, M is the number of genes that are annotated with certain GO terms, and m is the number of DEGs in M. A Bonferroni correction was used to calculate the p-value. A p-value ≤0.05 was considered significant in our study. GO terms fulfilling this condition were defined as significantly enriched GO terms among the DEGs. This analysis was utilized to determine the main biological functions that the DEGs perform.

Pathway-based analysis helped us to further understand the biological functions of the genes. The Kyoto Encyclopedia of Genes and Genomes (KEGG) database is the major publicly available, pathway-related database. The same calculation as that used in the GO analysis was used to determine significance: however, in this case, N is the number of genes with the KEGG annotation, n is the number of DEGs in N, M is the number of all genes annotated to a specific pathway, and m is the number of DEGs in M. The KEGG pathway analysis of the DEGs was performed using Cytoscape software (version 2.6.2) (http://www.cytoscape.org/) with the ClueGO plugin (http://www.ici.upmc.fr/cluego/cluegoDownload.shtml) [31].

### Optimization and Extension of DEGs

Because only the CDS of the denoted reference genes existed in the present *O. aries* genome and many of the sequences had imperfect ORFs, we optimized and extended these DEGs according to the sequencing data. After the reads had been aligned to the reference genome, the genomic regions with continuous reads and ≥2 uniquely mapped reads formed transcriptionally active regions. The different transcriptionally active regions were connected to form a potential gene model using the paired-end data. The extensions of the 5′ and 3′ boundaries were determined by comparing the potential gene model with the existing gene sequence.

### UTR Analysis of the DEGs

According to the extended and optimized results, we predicted the untranslated regions (UTRs) of the DEGs. If the gene had a complete ORF, we were able to determine the genes’ UTRs at one or both ends according to the extension results. If the ORF was incomplete, we predicted the UTR sequences according the sequence surrounding the initiation and termination codons in the extended region.

### Protein-protein Interaction Network Analysis of the DEGs

We converted the gene IDs of 1,300 DEGs to the gene symbols of the encoded proteins using bioDBnet software (www.biodbnet.abcc.ncifcrf.gov/db/db2db.php#biodb). We then mapped the gene symbols onto the human functional interaction network found in the Reactome database [32] using the Reactome FI network plug-in in the Cytoscape software [33].

### ORFs and UTRs Analysis of Novel Transcript Units

We used the hidden Markov model parameters of the sheep gene datasets to train the program to predict the complete ORFs and UTRs of the novel transcripts [34]. For the novel transcripts with complete ORFs, we predicted their corresponding protein sequence.

### Real-time Quantitative RT-PCR

To validate the reliability of the RNA-seq gene expression data, we performed qRT-PCR on 18 randomly selected differentially expressed genes. After transcription of 1 µg of total RNA into cDNA using the Transcriptor High Fidelity cDNA Synthesis Kit (Roche, No. 05081955001), qRT-PCR was performed using SYBR Premix Ex Taq II (TaKaRa, DRR081A), 7.5 µL of 2X SYBR Premix Ex Taq II reaction buffer, 0.3 µL of primers (0.4 µmol each), 0.3 µL of 50X ROX Reference Dye II, 1.5 µL of cDNA (10 pg/µL∼1 µg/µL), and RNase-free dH_2_O to a final volume of 15 µL. The reaction mixtures were incubated in a 96-well plate at 95°C for 30 sec, followed by 40 cycles of 95°C for 5 sec and 60°C for 30 sec using an Mx3000p SYBR Green real-time quantitative PCR analyzer (Stratagene, USA). All reactions were performed in triplicate. Following amplification, a melting curve analysis was performed to verify the specificity of the reactions using the Mx3000/Mx Pro software (Stratagene, USA). GAPDH was chosen as the reference gene for normalization of all the data because it was stably expressed [35], [36]. Gene expression ratios were normalized to the GAPDH expression in the same sample. The relative amount of each gene was calculated using the 2^−△△CT^ method [37] according to standard curve. The expression level of each gene was expressed as the 2^−△△CT^ mean ± SE. Fold was calculated for each gene by 2^−△△CT^ mean of DP/SH. By examining the significant difference among three repeats, a one-way ANOVA was used to determine repeatability of real-time PCR reaction, and differences were considered significant when p<0.05.

## Supporting Information

File S1 **Contains the files: Table S1.** Highly expressed genes (RPKM≥500) occurring in both libraries.xlsx. **Table S2.** Thirty-four DEGs related to muscle cell development and differentiation.xls. **Table S3.** KEGG pathway annotation of DEGs.xlsx. **Table S4.** The extension of DEGs according to RNA-seq.xls. **Table S5.** UTR prediction of the DEGs.xlsx. **Table S6.** Gene symbols for 1300 DEGs.xls. **Table S7.** UTR sequence of novel transcript units.xlsx. **Table S8.** Predicted UTR regions of novel transcript untis.xlsx. **Table S9.** Predicted protein sequences in the two library.xlsx. **Figure S1.** Gene expression changes in the ECM-receptor interaction. A red frame indicates genes that are up-regulated in the SH sheep compared to the DP sheep, and a green frame indicates genes that are down-regulated. **Figure S2.** Gene expression changes in the regulation of the actin cytoskeleton pathway. A red frame indicates genes that are up-regulated in the SH sheep compared to the DP, and a green frame indicates genes that are down-regulated. **Figure S3.** Experimental animals license.
(ZIP)
