# Supplementary figures and images for: Analysis of Differential Gene Expression and Novel Transcript Units of Ovine Muscle Transcriptomes
Source: PLoS One. 2014 Feb 26;9(2):e89817. doi: 10.1371/journal.pone.0089817 (PMC3935930; doi:10.1371/journal.pone.0089817)

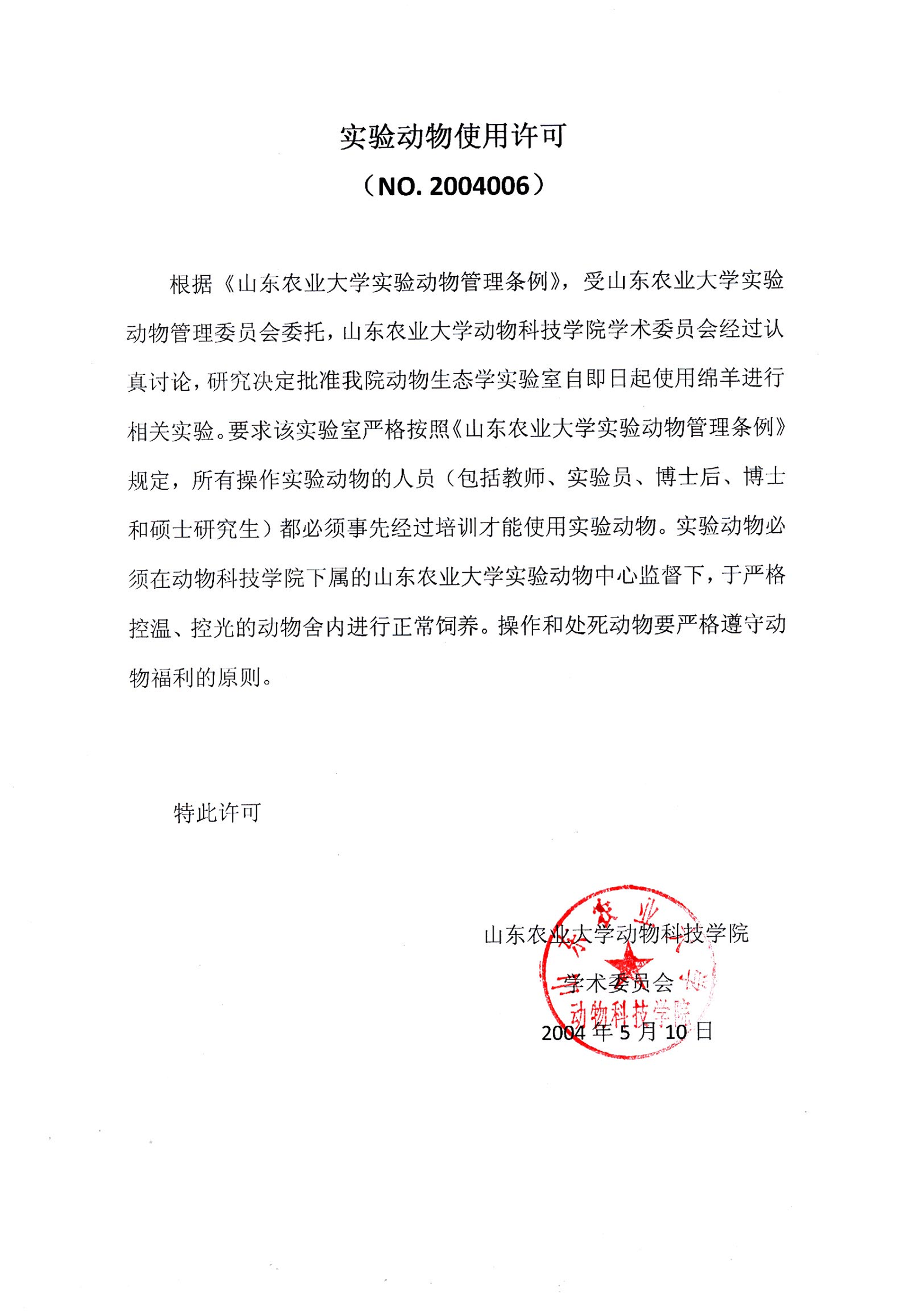

Supplement: File S1 — Contains the files: Table S1. Highly expressed genes (RPKM≥500) occurring in both libraries.xlsx. Table S2. Thirty-four DEGs related to muscle cell development and differentiation.xls. Table S3. KEGG pathway annotation of DEGs.xlsx. Table S4. The extension of DEGs according to RNA-seq.xls. Table S5. UTR prediction of the DEGs.xlsx. Table S6. Gene symbols for 1300 DEGs.xls. Table S7. UTR sequence of novel transcript units.xlsx. Table S8. Predicted UTR regions of novel transcript untis.xlsx. Table S9. Predicted protein sequences in the two library.xlsx. Figure S1. Gene expression changes in the ECM-receptor interaction. A red frame indicates genes that are up-regulated in the SH sheep compared to the DP sheep, and a green frame indicates genes that are down-regulated. Figure S2. Gene expression changes in the regulation of the actin cytoskeleton pathway. A red frame indicates genes that are up-regulated in the SH sheep compared to the DP, and a green frame indicates genes that are down-regulated. Figure S3. Experimental animals license. (ZIP) [file pone.0089817.s001.zip › Supporting information files/Fig S3. Experimental animals license.tif]

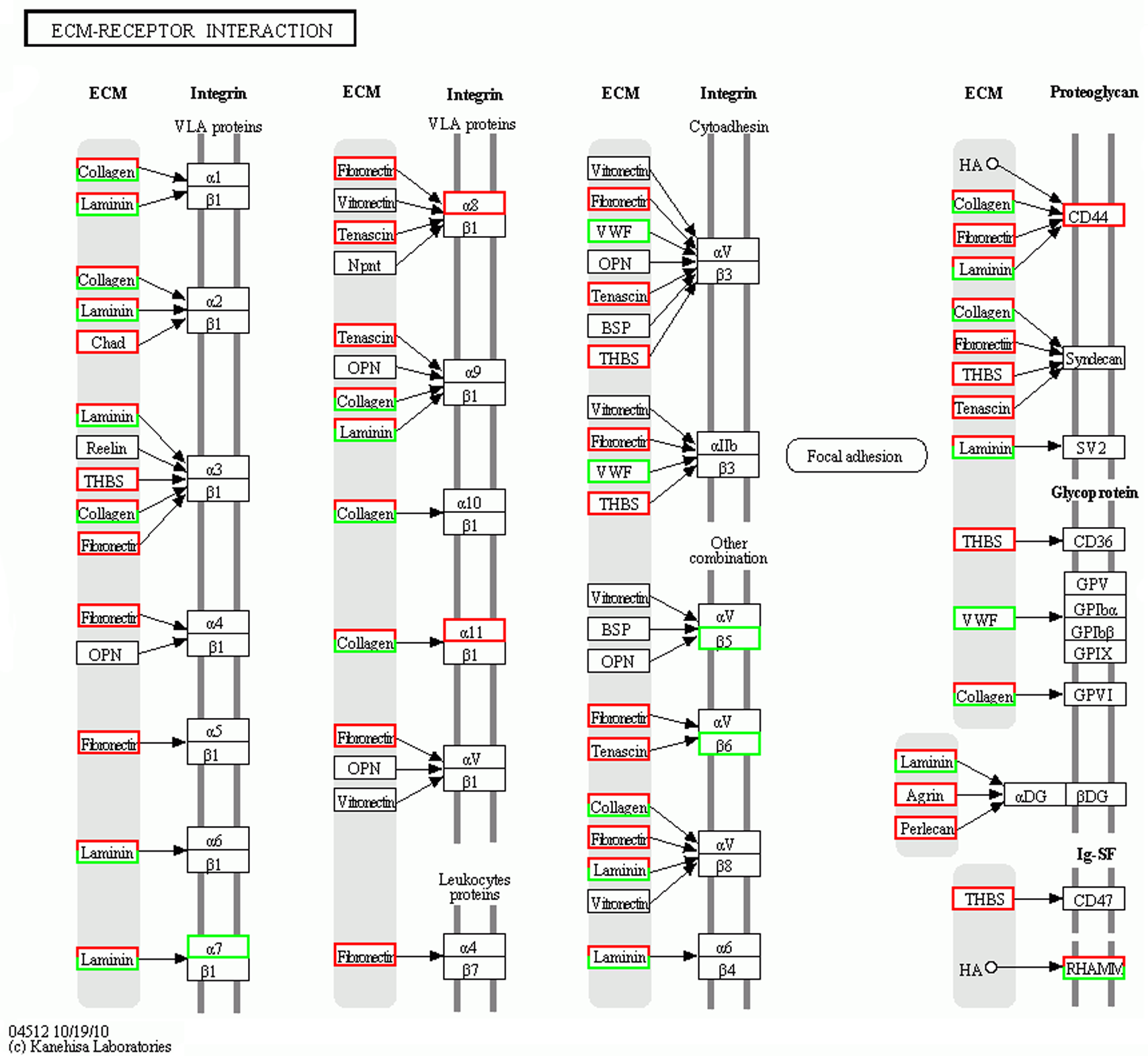

Supplement: File S1 — Contains the files: Table S1. Highly expressed genes (RPKM≥500) occurring in both libraries.xlsx. Table S2. Thirty-four DEGs related to muscle cell development and differentiation.xls. Table S3. KEGG pathway annotation of DEGs.xlsx. Table S4. The extension of DEGs according to RNA-seq.xls. Table S5. UTR prediction of the DEGs.xlsx. Table S6. Gene symbols for 1300 DEGs.xls. Table S7. UTR sequence of novel transcript units.xlsx. Table S8. Predicted UTR regions of novel transcript untis.xlsx. Table S9. Predicted protein sequences in the two library.xlsx. Figure S1. Gene expression changes in the ECM-receptor interaction. A red frame indicates genes that are up-regulated in the SH sheep compared to the DP sheep, and a green frame indicates genes that are down-regulated. Figure S2. Gene expression changes in the regulation of the actin cytoskeleton pathway. A red frame indicates genes that are up-regulated in the SH sheep compared to the DP, and a green frame indicates genes that are down-regulated. Figure S3. Experimental animals license. (ZIP) [file pone.0089817.s001.zip › Supporting information files/Fig. S1.Gene expression changes in the ECM-receptor interaction.TIF]

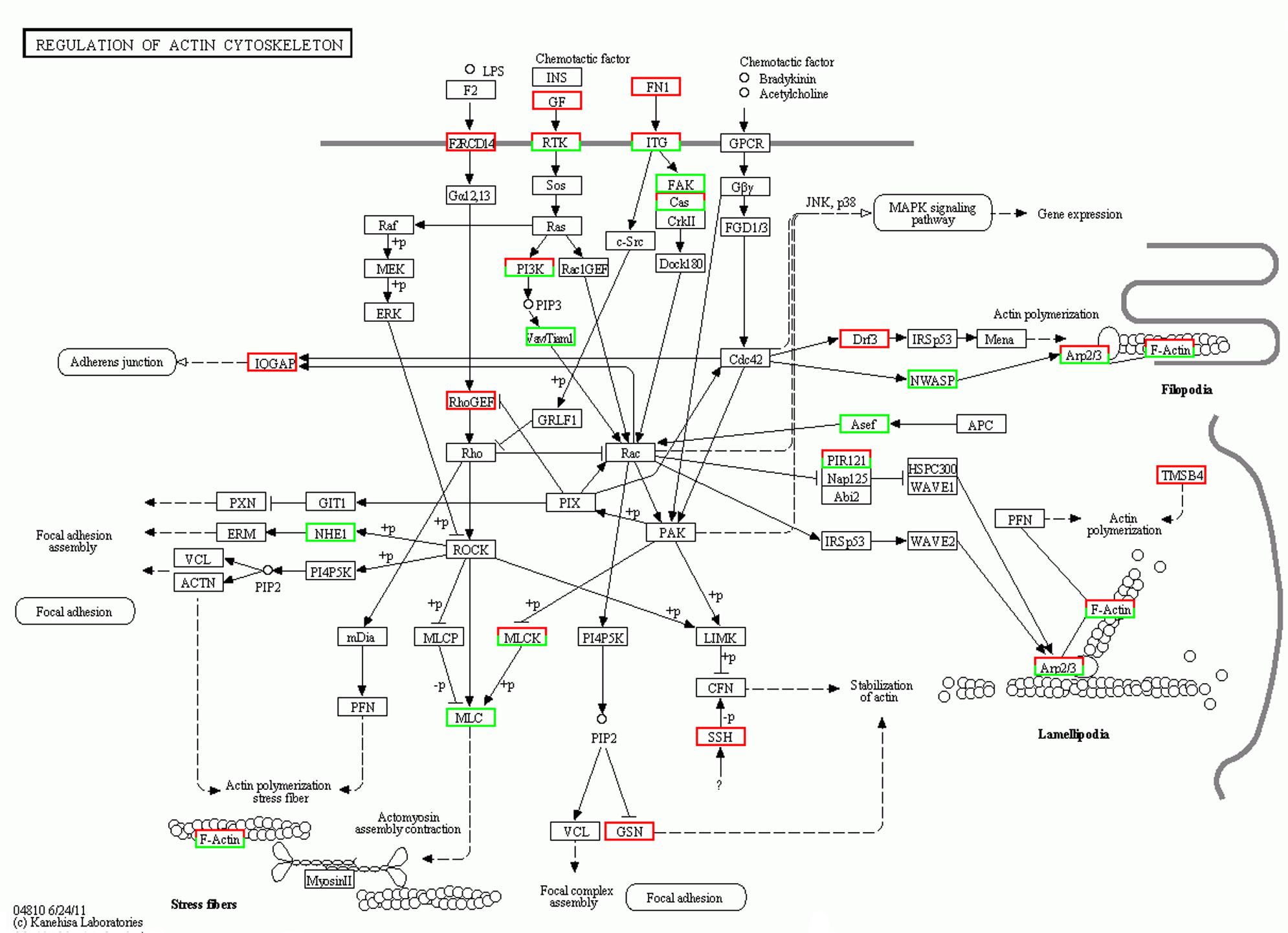

Supplement: File S1 — Contains the files: Table S1. Highly expressed genes (RPKM≥500) occurring in both libraries.xlsx. Table S2. Thirty-four DEGs related to muscle cell development and differentiation.xls. Table S3. KEGG pathway annotation of DEGs.xlsx. Table S4. The extension of DEGs according to RNA-seq.xls. Table S5. UTR prediction of the DEGs.xlsx. Table S6. Gene symbols for 1300 DEGs.xls. Table S7. UTR sequence of novel transcript units.xlsx. Table S8. Predicted UTR regions of novel transcript untis.xlsx. Table S9. Predicted protein sequences in the two library.xlsx. Figure S1. Gene expression changes in the ECM-receptor interaction. A red frame indicates genes that are up-regulated in the SH sheep compared to the DP sheep, and a green frame indicates genes that are down-regulated. Figure S2. Gene expression changes in the regulation of the actin cytoskeleton pathway. A red frame indicates genes that are up-regulated in the SH sheep compared to the DP, and a green frame indicates genes that are down-regulated. Figure S3. Experimental animals license. (ZIP) [file pone.0089817.s001.zip › Supporting information files/Fig. S2. Gene expression changes in the regulation of the actin cytoskeleton pathway.tif]
